# Tracing State Structure for Ecological Processes in Soil Including Greenhouse Gas Exchange with Lower Atmosphere †

**DOI:** 10.3390/s24113507

**Published:** 2024-05-29

**Authors:** Miki Sirola, Markku Koskinen, Tatu Polvinen, Mari Pihlatie

**Affiliations:** Environmental Soil Science, Department of Agricultural Sciences, Faculty of Agriculture and Forestry, Institute of Atmospheric and Earth System Research, University of Helsinki, P.O. Box 56, FI-00014 Helsinki, Finland; markku.koskinen@helsinki.fi (M.K.); tatu.polvinen@helsinki.fi (T.P.); mari.pihlatie@helsinki.fi (M.P.)

**Keywords:** principal component analysis, visualization, state discovery, soil and flux data, ecological process model

## Abstract

Exploring data aids in the comprehension of the dataset and the system’s essence. Various approaches exist for managing numerous sensors. This study perceives operational states to clarify the physical dynamics within a soil environment. Utilizing Principal Component Analysis (PCA) enables dimensionality reduction, offering an alternative perspective on the spring soil dataset. The K-means algorithm clusters data densities, forming the groundwork for an operational state description. Soil data, integral to an ecosystem, entails evident attributes. Employing dynamic visualization, including animations, constitutes a vital exploration angle. Greenhouse gas variables have been added to PCA to achieve more understanding in the interconnection of gas exchange and soil properties. Pit data and flux data are analysed both separately and together using a data-driven approach. The results look promising, showing the potential to add new values and more detailed state structures to ecological models. All experiments are conducted within the Jupyter programming environment, utilizing Python 3. The relevant literature on data visualization is examined. Through combined techniques and tools, the potential features of the soil ecosystem are observed and identified.

## 1. Introduction

Data exploration serves the purpose of comprehending both the dataset and the system itself, particularly when dealing with a multitude of sensors. This paper adopts an approach that focuses on delineating operational states and dynamic behaviours within the system. Dimensionality reduction offers an alternative perspective on the data, facilitating two- or three-dimensional visualizations. The Principal Component Analysis (PCA) method is employed for this purpose, where states are derived from identifying data densities or clusters through the K-means algorithm.

The initial methodological concept used in this article is partly developed in our earlier work [1]. This study is an extension of the work [2], where pit soil data is analyzed, and process states are discussed. This article aims to define states and explore transitions to enhance understanding of the ecosystem. The exploration specifically involves soil pit data, looking for physical interpretations of the defined states for a comprehensive system understanding. Here, we also add the exchange of greenhouse gases to the analysis and explore what kind of effect it has to the defined state structure. Both static and dynamic visualization methods, including animations, are employed to illustrate state compositions and transitions. The experiments in this article are implemented using the Jupyter programming environment and the Python 3 programming language.

The objective is to constitute the means to define states in ecological soil processes during different seasons. The hypothesis is that such physically supported modes can be found. With selected methodologies and used tools we have built groundwork for further steps in constituting an expressive state structure for soil processes. The scientific question is in better understanding the ecological processes in soil by determining process states with a data-driven approach.

This paper follows a structured organization, beginning with the definition of the problem and an introduction to the background, methods, and tools in Section 1. Section 2 deals with the related literature on visualization, while Section 3 presents the methods and tools. Section 4 introduces the soil pit data combined with flux data, and the core of the paper, including state discovery exploration and visualization results, is detailed in Section 5. The Discussion (Section 6) and Conclusion (Section 7) summarize the key contents before the reference list.

## 2. Related Work

Various perspectives on data visualization are explored in the literature through the following references. The combination of visualization and machine learning in the context of data centre management is thoroughly investigated and documented in [3]. Additionally, the relationship between visualization and machine learning is discussed in [4]. However, there is a notable absence of corresponding studies focusing on soil data.

An interesting inquiry lies in the examination of how Principal Component Analysis (PCA) techniques have been applied in animation research. While the primary focus of this paper is on data exploration, a broader perspective is also within the scope of our investigation. Notably, PCA methods are commonly employed in facial animation, as evidenced by references [5,6,7,8]. These sources focus on the application of PCA in facial animation, covering areas such as shape correctives, parametrization of mouth images, and reduction of parameters in three-dimensional facial animations.

Reference [3] introduces a novel tool for data centre management, incorporating data visualization and data management capabilities. In [4], a design space for the visualization of multidimensional comparative data analytics is outlined.

The application of PCA in animation research is further explored in several references. These sources cover many topics, including compression techniques in 3D animations [9], geometry compression [10], soft-body 3D animations [11], and virtual human motion animations [12,13]. Paper [14] presents an affine transformation matrix in PCA to compress data in 3D animation models.

Recent studies, as outlined in [15,16,17], continue to employ PCA in diverse contexts such as the compression of 3D mesh animation data, object-based compression of three-dimensional animation geometry, and motion decomposition in computer animation.

In the domain of character animation, reference [18] discusses the utilization of PCA in a reduced linear regression problem, while references [19,20] explore a matrix and tensor-based approximation of 3D face animations and colour quantization methods, respectively, using PCA techniques. PCA–based compression techniques are also discussed in [21].

The applicability of PCA extends to broader domains, as evidenced by references [22,23]. In [22], PCA is utilized in a 3D city model generalization for an electricity simulation, while [23] employs the Hidden Markov Model (HMM) and Deep Neural Network (DNN) techniques in synthesizing facial animation, comparing the results with conventional PCA for objective evaluation.

## 3. Methods and Tools

The research in this study employs various methodologies, including data exploration, statistical analysis, visualization using selected assistant methodologies, and the creation of exploration animation prototypes.

The primary assistant methodology utilized is Principal Component Analysis (PCA) [24], a technique that condenses N variables into a defined number of projections. Typically, the first two or three components are employed in visualization, with the first component containing the most variance in the data, followed by the subsequent components. While dimensionality reduction inevitably leads to some information loss, it simultaneously provides a completely new perspective on the data.

PCA effectively describes the structure, properties, states, and state transitions of the data. Beyond examining the variance distribution for each component, it allows an assessment of how much each variable influences each PCA component, known as PCA loadings. An interesting view to PCA is presented in [25]. 

Another crucial assistant methodology is the K-means clustering algorithm [26], which highlights local accumulations and densities in the data. Despite its sensitivity to outliers, the K-means algorithm is generally reliable and stable, effectively revealing data densities, clusters, and illustrative state transitions.

In addition to conventional PCA visualizations, dynamic behaviour in the data is illustrated using PCA animations. These animations vividly portray the composition of states over time, providing descriptive explanations of state transitions. They prove valuable in identifying anomalies in the data, sometimes enabling the recognition of failure states.

Throughout all implementations and experiments in this paper, the Jupyter tool version 6.4.8 and programming environment, along with the Python 3 programming language, are consistently employed. Known Python libraries are used in the implementation. From “sclearn.decomposition”, “PCA” is imported for PCA, and from “sclearn.cluster”, “KMeans” is imported for grouping. In addition, libraries such as “pandas”, “numpy”, “matplotlib”, and “seaborn” are used, and some others.

## 4. Data

The provided data are based on soil observations taken during the spring term of 1.4.23 to 31.5.23 originating from the Viikki measurement station situated in Helsinki. In addition, some greenhouse gas concentrations and fluxes from the same period are combined with the soil data. This data production is a part of the research conducted by the INAR (Institute for Atmospheric and Earth System Research) in Finland, which is affiliated with a widespread international network of research stations [27]. The specific measurement station, SMEAR-Agri, focuses on capturing soil characteristics within an agricultural field environment, in this case during the spring season. Modern measurement technology has been used in the data acquisition process [28].

The soil dataset includes various variables, including Electrical Conductivity, Redox Potential, Water Content, Temperature, Matric Potential, and Water Level. These variables, except for Water Level, are measured at five separate depth levels below the soil surface: 14 cm, 36 cm, 60 cm, 87 cm, and 123 cm. With a total of 17,856 measurement samples and 47 variables, only the initial 33 variables are utilized in the analysis due to their higher significance. Some of the excluded variables are of lesser importance and contain binary data unsuitable for Principal Component Analysis (PCA) examination. From the flux dataset from the same measurement station, eight variables have been selected to complete the analysis. The variables are CO_2_, N_2_O, CO, and H_2_O concentrations and fluxes.

**Figure 1:**
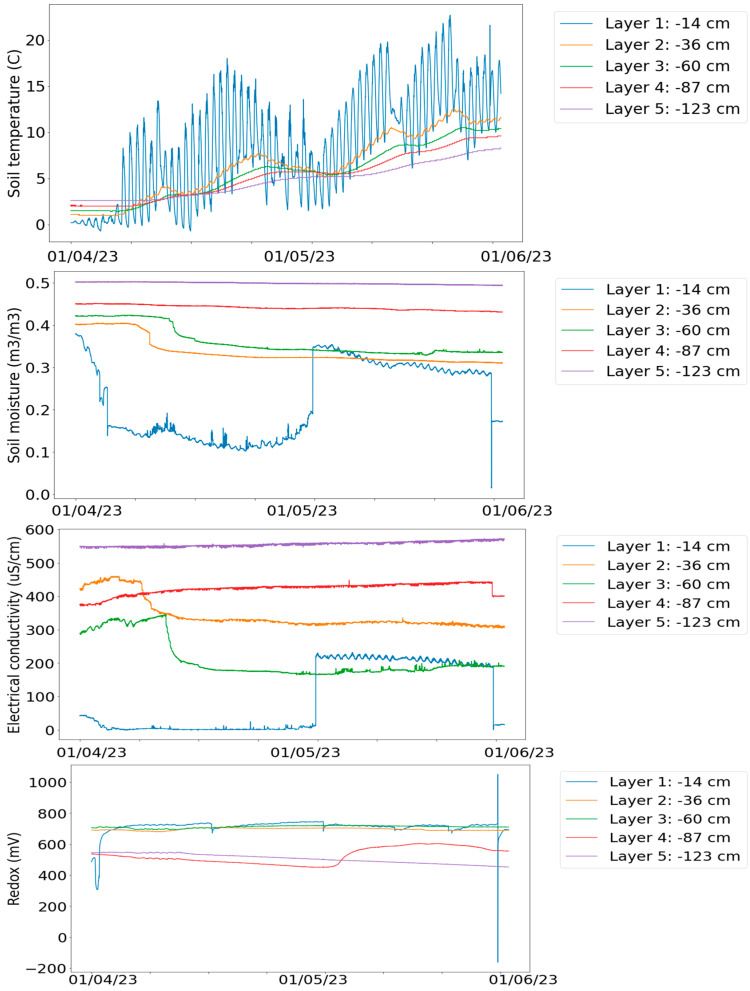
Some main variables in soil pit data during the spring term, 2023. The unit of soil moisture is expressed as the water content ratio (0 completely dry, 1 completely wet).

**Figure 2:**
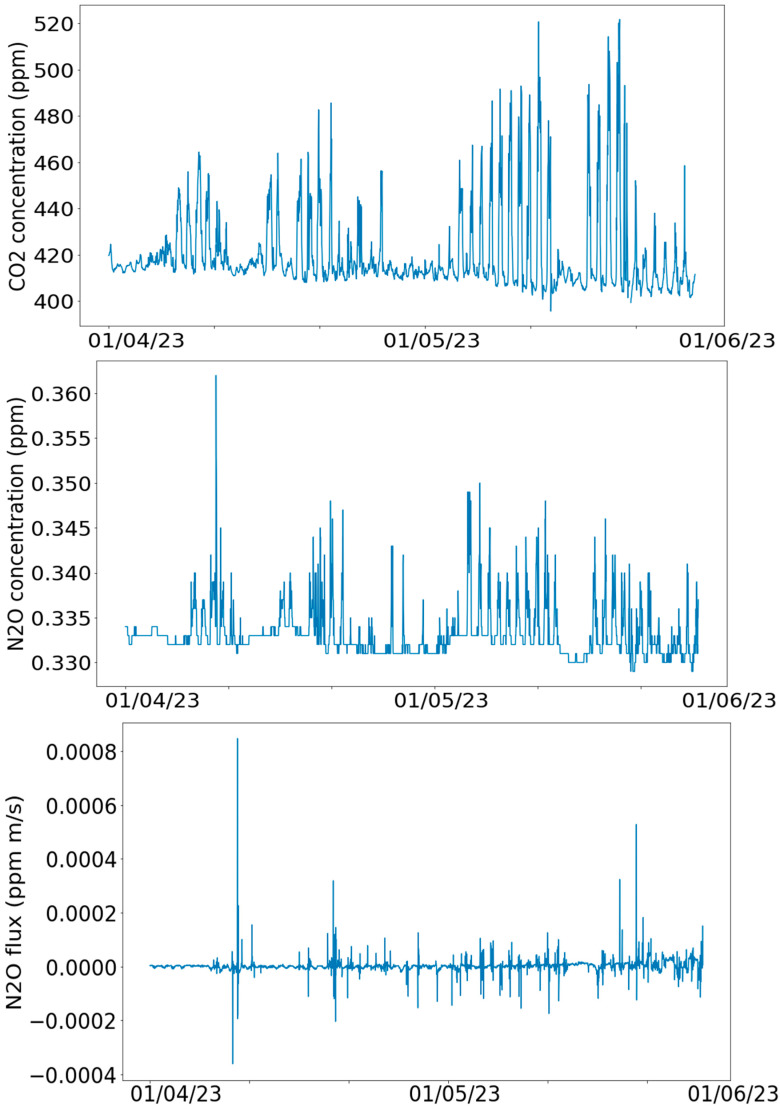
Example variables in greenhouse gas flux data during spring term, 2023.

Figure 1 illustrates the variations of Temperature, Water Content, Electric Conductivity, and Redox Potential across all five depth levels. Figure 2 illustrates concentrations of the greenhouse gases CO_2_ and N_2_O and N_2_O flux.

In the beginning of April, the ground is still frozen, and the surface temperature is the lowest compared to the deeper level. When the spring term proceeds, the order changes; see Figure 1. When the ground has melted, the layer nearest to the surface varies the most diurnally. The sudden changes in soil moisture surface level are due to heavy rain, and long dry periods combined with high day temperatures may cause the opposite effect at some point. Generally, the changes in surface variables are faster than those deeper in the ground. Note that the data are not cleaned, so there may be error values seen as extra spikes, for instance.

## 5. State Discovery Exploration and Visualization Results

First, we analyze the pit dataset only from the spring term of 2023. In [2], we conducted a somewhat similar analysis for the data in the spring term of 2022, approximately at the same time of the year. There are many similarities in these two datasets, but also remarkable differences. 

**Figure 3:**
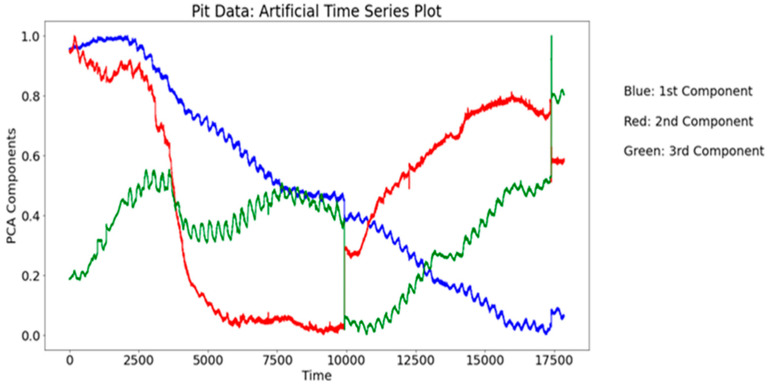
The three first PCA components in the analysis; artificial time series expression.

**Figure 4:**
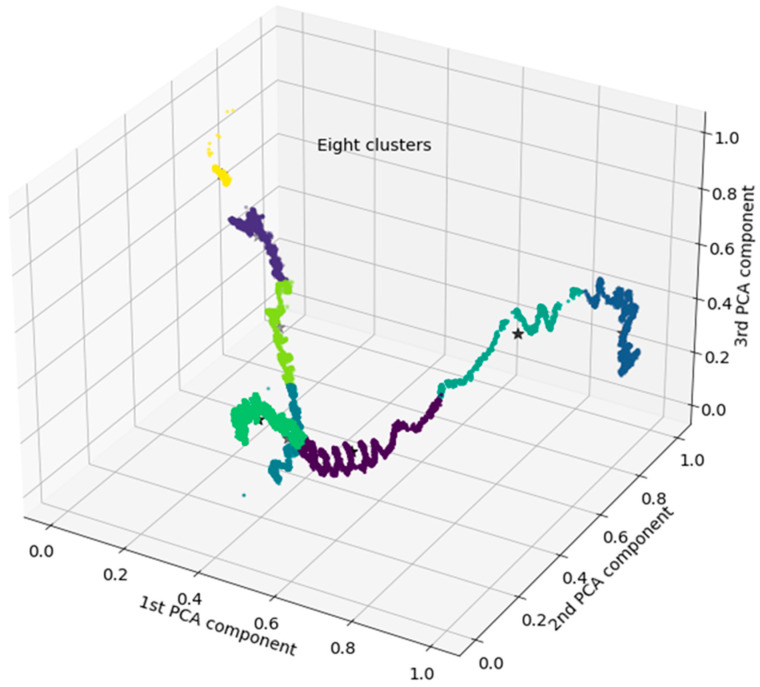
A three-dimensional scatter plot of the PCA result of pit data, grouped into eight clusters.

**Figure 5:**
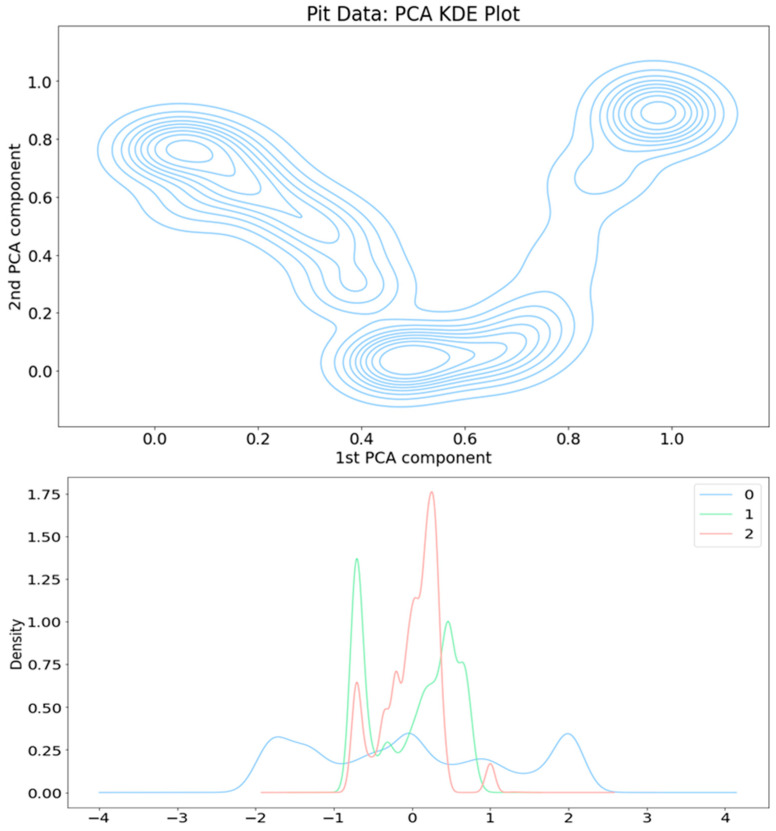
The density function formed as a KDE plot (upper plot) and PCA component curves (lower plot). In the lower plot, the first PCA component is in blue, the second PCA component in green, and the third PCA component in red.

The PCA reduces the primary aspects in this ecological process into three core factors; see the artificial time series representation in Figure 3. The spatial pattern of these three components is seen in Figure 4 The distribution function illustrations in Figure 5 introduce an alternative perspective to the same dataset.

In the first PCA component (shown in blue in Figure 3), the ground temperatures are the predominant factors. In the second PCA component (shown in red in Figure 3), soil moisture and the closely associated electrical parameters are predominant. In the third PCA component (shown in green in Figure 3), the groundwater level and associated quantities prevail.

Two distinct representations of distribution functions in the PCA are shown in Figure 5. In Figure 4, there is a 3-dimensional scatterplot of the PCA outcome, clarifying clustering information, divided into eight clusters.

PCA loadings (refer to Table 1) exhibit the predominance of each variable in each PCA component. The table also presents the variance distributions for the first three PCA components. In this assessment, the first PCA component has considerable dominance, capturing 74% of the total variance, while the subsequent components capture 13% and 6%, respectively. The table identifies the most influential variables in each of the first three PCA components.

**Figure 6:**
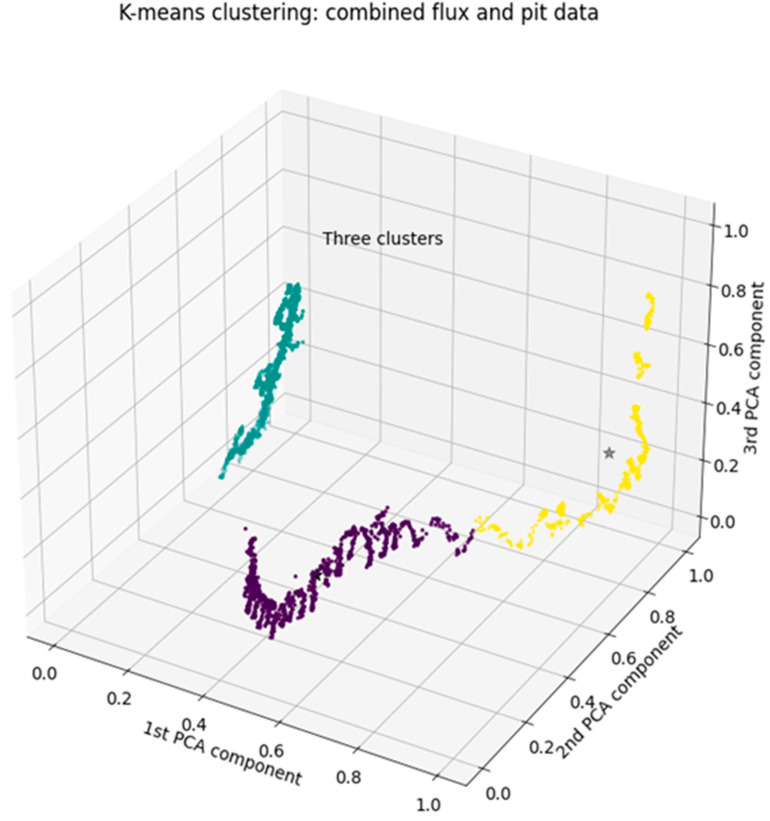
The three-dimensional scatter plot of the PCA result of combined pit and flux data, grouped into three clusters.

K-means clustering serves as the basis for defining the states in this process. In Figure 4, eight states are recognized, while in Figure 6, only three states are identified. A state represents a clear physical expression of the process. However, determining the number of states and their interpretations is not straightforward.

**Figure 7:**
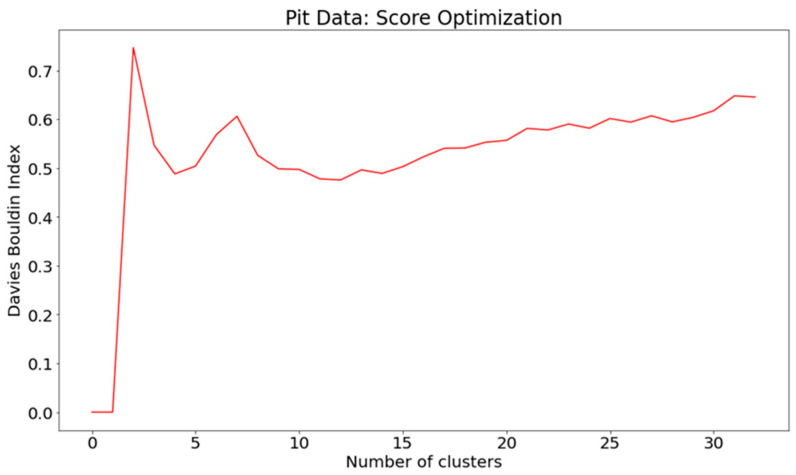
Score optimization by Davies–Bouldin index.

The distribution functions, particularly the KDE plot, in Figure 5, clearly supports three states, while the Davies–Bouldin Index favours eight clusters as well but also three states as a global maximum, as shown in Figure 7. Eight clusters represent a clear local maximum in the score optimization.

Naming three states is relatively straightforward, but in an eight-state interpretation, assigning different meanings to each state becomes challenging. The soil samples are collected during the spring term in Finland; thus, the chronologically initial state is evidently frozen ground (ground frost). The subsequent state is melting ground, and the third state melted ground. After the ground melts, temperatures begin to gradually increase.

After melting, soil moisture rises, electrical conductivity in the soil increases, and initially, the water level rises, but subsequently decreases as melting completes and temperatures rise further. Electrical parameters exhibit a strong correlation with soil moisture.

The remaining states emerge from variating water content values due to alternating wet and dry periods. Here, electrical variables strongly correlate. Thus, in an eight-state interpretation, one may question whether each new state truly represents a novel condition or if it represents previous states to some extent. Notably, the gradual rise in temperatures makes a clear difference between potentially repetitive states.

The state transitions follow a chronological order due to the spring term data, with summer approaching. However, this temporal sequence is not universal; for instance, considering data over a full year would reveal a cyclical state structure, including a return to frozen ground conditions.

It is important to note that soil moisture initially increases rapidly after the ground melts but later decreases. Additionally, there is a temporary moisture increase in late spring.

In summary, key factors driving state transitions include frozen ground, melting ground (with significant changes across multiple variables), temperature changes, and variations in soil moisture. Electrical parameters also play a crucial role, closely tied to soil moisture levels.

This type of analysis can aid in identifying failure states as well, although in this dataset, failure states are not applicable, as all states represent real ecological processes. Measurement errors could potentially appear as failure states, but no such anomalies are evident in this dataset.

Similar analysis was done for the combined dataset, where eight flux variables were added to the dataset as additional columns. The results were somewhat similar with some minor deviations. We are not sure about the reason for the slightly weaker appearance of the flux variables in the analysis than what was expected beforehand.

Compared to Table 1, where we see the PCA loadings of pit data only, a few additional effective variables are recognized. Relative variances for the first three PCA components are now 0.71, 0.13, and 0.06. In the first PCA component column in Table 1, WC2 and WC4 are replaced by WC5. In the third PCA component column MP1 (matrix potential in layer 1) and H_2_O concentration should be added (both > 0.2).

If we compare the results to Figure 3, the biggest difference is in the third PCA component, which seem to turn into a kind of mirror image (jumping up instead of down). The density functions look surprisingly similar in both analyses. In the Davies–Boulding Index (see Figure 7), there is also a clear peak in three clusters, and a local maximum in eight clusters, but with the flux variables, the local maximum is recognized as being much milder.

In Figure 6, the PCA result for combined pit and flux data as a three-dimensional scatter plot grouped into three clusters is seen. Compared to Figure 4—pit data only—the shape in this visualization result of the combined data is somewhat similar, with some notable differences.

Because the differences in the combined data including flux variables compared to only pit data were somewhat milder than expected, we have carried out some more exploration and analysis for a dataset where we examined these eight additional flux variables alone. This was done to find out some important properties in the flux data that remain a bit hidden in the combined analysis.

The structure of the flux data is very different from the previous ones, and we explain here some of the most important points to complement our analysis. All previously presented visualizations look very different with this dataset, but we describe only a few. As in the previous analysis, we could not fully understand the minor weights of flux variables; here, we recognized a similar effect with weight points in fluxes compared to concentrations of the greenhouse gases, which remain now more in the background. All variables are normalized in all our PCA analyses.

For pure flux data, the relative variance is 0.45 for the first PCA component, 0.30 for the second PCA component, and 0.12 for the third PCA component. Variables (concentrations) of C_H2O_ have strong dominance in the first PCA component, and C_CO2_ has strong dominance in the second PCA component. In the second PCA component, C_N2O_ and w’CO_2_*(carbon dioxide flux) also have remarkable influence, the latter by reverse means (also marked here as *). In addition, C_CO_* in the first PCA component; C_CO_, C_H2O_*, and C_N2O_* in the second PCA component; and C_CO2_, C_CO,_ and C_N2O_ in the third PCA component have effects > 0.2 (notice here also the asterisk, meaning reverse means).

**Figure 8:**
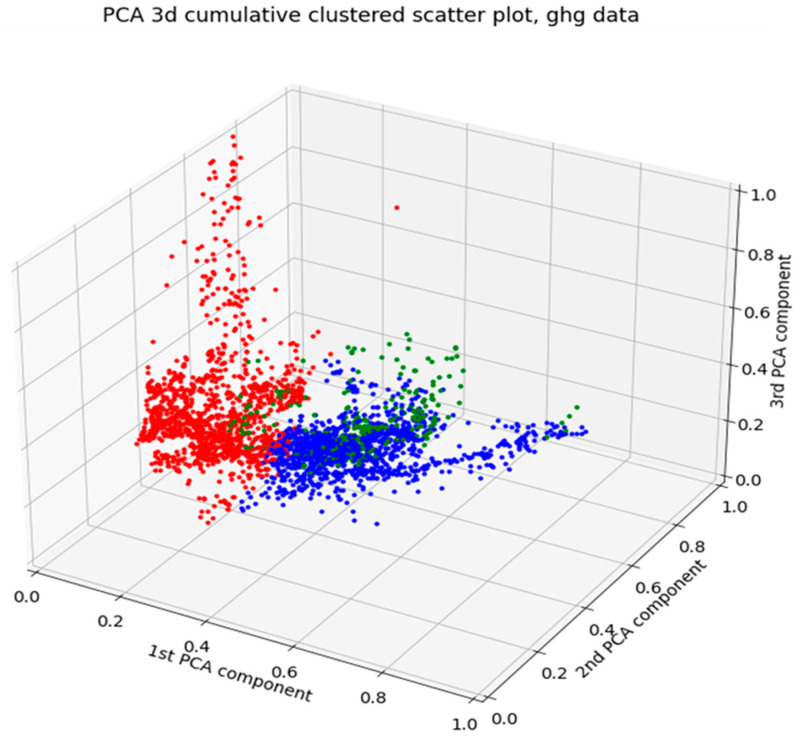
Exploration animation of pure flux variable PCA helps in state discovery.

Also, in this pure flux analysis, the Davies–Bouldin Index shows a clear global peak in three clusters, so three states are chosen for analysis here as well; see Figure 8. Because here the states do not follow each other similarly in chronological order, as in both previous analyses presented in this article, it is well justified to make an exploration animation to also check the state transitions in more detail.

**Figure 9:**
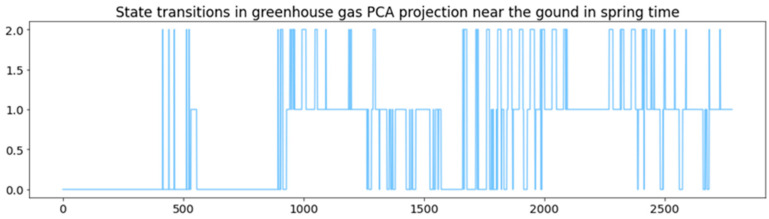
State transitions in pure flux data.

**Figure 10:**
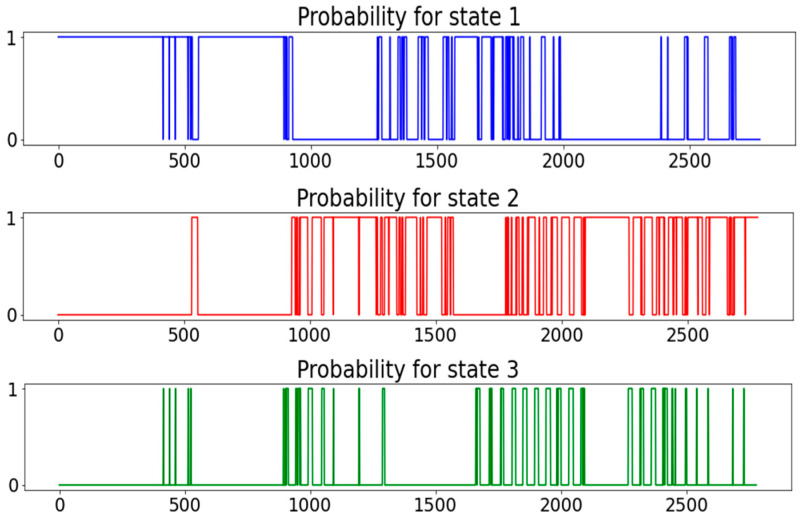
State probabilities for each state in pure flux data analysis.

To clarify the state transitions here even more, in Figure 9, the statistical state transitions in the same analysis are shown, and, finally, in Figure 10, the probabilities of each state at each moment during the period of examination.

In our prior publications [1,2], we highlighted animations as practical and effective tools for exploring and understanding states, thus providing physical interpretations of the process. Figure 8 showcases an animated structure using the pure flux dataset including only eight chosen variables from it and illustrating three distinct states, this time not clearly in chronological sequence. This figure represents a statistical summary of the dynamic animation.

The animation presented in Figure 8 begins from the red cluster and ends at the blue one. This animation spans all 2780 measurement samples in fifty-six seconds. The technical implementation offers adjustable parameters to control animation speed. The clustered PCA outcome serves as an illustrative scenario, highlighting some spring term characteristics derived from flux measurements.

Note that here the three states do not represent so clearly the three states in the previous analyses. So, this analysis encourages us to search for more explanations for more states than the three obvious ones: frozen ground, melting ground, and melted ground. To really constitute an ecological model physically explaining more states, we would need to understand and go deeper in the physical effects and chemical reactions in soil, especially in springtime, but of course more seasons could be added to the analyses as well. In this kind of data-driven analysis, we will not get that far.

What we can see already in this analysis that, in spring, when soil melts, a lot of things begin to happen underground, including chemical reactions, producing greenhouse gases. But to constitute clear, new, named states, we need a lot more work, including better physio-chemical understanding and knowledge. 

## 6. Discussion

This article aims to illustrate the functionality of exploratory state discovery and visualization techniques, particularly with soil pit data, and flux data combined with it. The animated presentation of clustered PCA outcomes offers insights into the temporal evolution of various operational states within an ecological process.

An extensive literature review investigates visualization techniques and animation applications, revealing numerous instances across different applications, but none specifically with soil data or other nearby measurement types, such as flux data.

The methodologies and tools employed are standard and widely recognized. PCA effectively clarifies the data structure, forming a solid foundation for identifying system states. K-means clustering aids in identifying data densities and grouping structures, which can be named operational states. Prediction methods are not utilized in this study. All experiments and realizations utilize the Jupyter programming tool and Python 3 language.

Defining states within an ecological system is challenging, particularly considering variables affected by weather variations, which are not easy to be used in achieving reliable predictions. Operational states, however, offer insight into system understanding and related phenomena.

Static visualization is simpler than dynamic visualization with animations, which are challenging to describe in a scientific paper limited to text, statistics, tables, and static figures. The objective is to present these concepts as clearly as possible within this format.

Various data normalization scalers are available, with the min–max scaler chosen for this analysis. While standard scaler is often preferred, the min–max scaler works well with these datasets, not being affected so much by outliers.

No failure states were identified in this analysis due to precise measurements. However, investigating measurement error states, such as an example involving a Redox sensor cable being severed, could offer valuable insights for future research.

In [2], the analysis was conducted with soil data in spring 2022. Here, we have performed a similar analysis for spring 2023 pit data. In the analysis, many similar features can be noticed, but also some remarkable differences. In both cases the score optimization (see Figure 7) gives a strong global maximum in three clusters and supports our three-state hypothesis of soil states in spring term (frozen ground, melting ground, and melted ground).

But now in spring 2023, we get a strong local maximum in eight clusters (instead of seven in spring 2022), but now the KDE density function (see Figure 5, upper plot) strongly supports only the three-cluster model (2022 spring data supported here by the seven-cluster model). In component–curve density plot (see Figure 5, lower plot) more states could be identified. The PCA loadings table looks rather similar, with some remarkable changes. Many of those differences can be explained by interpreting the artificial time window approach (see Figure 3), where now the first component is decreasing (instead of increasing in the 2022 data analysis), and the third component makes a kind of mirror image turn in the middle of the spring term period.

Compared to the previous analysis in [2], we have also expanded our analysis by adding eight flux variables to the pit dataset. While carrying out this analysis, it was noticed, though, that the flux variables had less influence on the result than expected. The reason for the rather weak weighting in some of the flux variables is not known. With flux variables, a not very strong local maximum was found in the score optimization.

Still, it looks promising that flux variables could help us to define more states in this ecological process, especially while the ground has melted. To understand these phenomena more, we repeated the analysis for the eight flux variables only. To conduct a thorough analysis with the state transitions, an exploration animation of the PCA result, including clustering, was performed. Here it was noticed that the states do not now only follow each other in chronological order, as in both previous analyses (and in the 2022 spring data analysis as well).

To define more than three states and explain them, we need to go deeper in the physiochemical analysis, mostly in summertime, or at least during the melted ground period. For instance, there exists knowledge about the behaviour of Redox variables in soil connected to chemical reactions and the resulting greenhouse gas releases [29]. Certain Redox potential value ranges can be connected to certain ions appearing in soil, and the chemical reactions producing certain greenhouse gases are known.

But already, our analysis here reveals the great potential for these kind of further steps in building up better ecological models. Our analysis includes a description of the state transitions both by animated scatter plot and direct state transition plot, and the probabilities of each of the three defined states through the whole period. These tools were not really needed in our earlier cases, where all states followed each other in chronological order. However, in this study, we do not try to carry out any predictions, only classifications of state-based characteristics. Based on our earlier studies [1], we could use, for instance, the Hidden–Markov model [30] to predict states in future. The bookkeeping of farming field activities could be utilized as well.

Our analysis here does not consider wind directions. The assumption is that the other fields nearby are similar enough for the accuracy in this analysis. In calculating real fluxes, the data are filtered according to different wind directions (coming to the Eddy–Covariance measurement point from different field blocks and analysed separately). A more thorough analysis here could be justified, as well as looking at the effects of each greenhouse gas separately. Now, our PCA combines all variables in one full description.

We have not found studies where similar methods would have been used with soil data or ecological systems in general. Therefore, it is difficult to find studies for scientific comparison. It may underlie our unique perspective of initial steps in the state models of ecological processes. In our literature review, the animation studies have some weak connection points to our perspective, and references [3,4] discussing data centre management relate to our earlier studies’ application domain.

While the methodologies and tools are conventional, this paper’s novelty lies in their application to a new domain. Additionally, the utilization of animations for data exploration offers a unique perspective. Quantitative assessment and comparisons with similar studies are challenging, given the qualitative nature of the benefits of this research.

## 7. Conclusions

This study demonstrates the effectiveness of exploratory data analysis in observing ecological systems by defining state structures that capture significant phenomena and employing visualization techniques, including animated grouped PCA results. These animations define the formation of states and their transitions over time, focusing on the spring season in agricultural soil.

The contribution of this research lies in its detailed case study, which highlights fundamental symptoms characteristic of such structures within the domain. The animated PCA proves to be a valuable tool for observing complex details in this specific context.

Adding greenhouse gas variables to the soil pit data analysis looks like a promising approach in building better ecological models in the future. Our data-driven study could gain from more detailed physiochemical analysis in soil.

## Figures and Tables

**Table 1:** PCA loadings for pit data.

	1st PCA Component	2nd PCA Component	3rd PCA Component
Relative variance	0.74	0.13	0.06
Pca.components_			
>0.4		WC_1-3_, EC_1-2_	WC_1_ *, EC_1_ *
>0.3		EC_3_	RX_2_ *
>0.2	RX_5_, EC_1_ *, WC_2-4_, T_2-5_ *	RX_2_ *, RX_4_	RX_3_ *

WC = Water Content; RX = Redox Potential; EC = Electrical Conductivity; WL = Water Level (Un-derground); MP = Matric Potential; T = Temperature; * = −(reverse correlation); indices 1-5 = layers in the ground.

## Data Availability

Dataset available on request from the authors.
